# Predicting miRNA–Disease Associations Based on Contrastive Learning of Linear and Nonlinear Features

**DOI:** 10.3390/genes17101246

**Published:** 2026-10-09

**Authors:** Yiming Sun, Weihua Zhu, Lei Li

**Affiliations:** 1 Department of Basic Medical Sciences, Clinical College of Anhui Medical University, Hefei 230031, China; yiming340@126.com (Y.S.); zhuweihua@aycc.edu.cn (W.Z.); 2 School of Artificial Intelligence, Anhui University, Hefei 230601, China

**Keywords:** miRNA–disease associations, singular value decomposition, graph autoencoder, contrastive learning, highway network

## Abstract

**Background:** MicroRNAs (miRNAs) regulate biological processes and are associated with human diseases. Computational prediction can prioritize candidate associations for experimental investigation, but many existing methods emphasize nonlinear representations and may underuse complementary linear information and interactions between miRNA and disease features. This study aimed to integrate these complementary representations for miRNA–disease association prediction. **Methods:** We developed Singular Value Decomposition and Graph Autoencoder for miRNA–Disease Association prediction (SGMDA). Singular value decomposition extracted linear representations from the association matrix, while graph autoencoders learned nonlinear representations from integrated miRNA and disease similarity graphs. Contrastive learning aligned the two representation spaces, and a highway network modeled interactions between the fused miRNA and disease features. A random forest classifier generated association scores. Evaluation included pair-wise five-fold cross-validation, global leave-one-out cross-validation, miRNA-wise and disease-wise cross-validation, ablation experiments, and case studies of lung and prostate neoplasms. **Results:** The reported comparisons showed competitive predictive performance across the evaluated settings, with lower performance under entity-wise cross-validation than under pair-wise cross-validation. Ablation experiments supported the complementary contributions of linear and nonlinear features, contrastive alignment, and highway-based feature interactions. Many highly ranked case-study candidates were supported by at least one external database. Temporal validation using different HMDD releases provided additional retrospective evidence for prioritizing subsequently recorded associations. **Conclusions:** Combining linear and nonlinear representations with contrastive alignment and feature-interaction modeling provides a useful framework for prioritizing potential miRNA–disease associations. Database support does not establish biological causality or constitute strict independent validation, and the predicted associations require further experimental investigation.

## 1. Introduction

With the rapid development of sequencing technology, researchers have discovered that miRNAs are one type of vital short noncoding RNA (22 nt) molecules that regulate gene expression at the post-transcriptional level [1,2,3,4]. Increasing studies have demonstrated that miRNAs play important roles in various complex biological processes, such as cell proliferation [5], differentiation [6], metabolism [7], and apoptosis [8]. Furthermore, substantial evidence has indicated that miRNAs are related to the development and progression of various human diseases, including lung, breast, and heart diseases [9,10,11]. Therefore, observing underlying miRNA–disease associations helps us to understand the molecular mechanisms of complex diseases, and thereby improve the accuracy of disease diagnosis and treatment. Extensive traditional experiments, which are limited in scale, time-consuming, and costly, have been conducted by researchers to observe the connections between miRNAs and diseases [12,13]. It is now necessary to develop computational models that can accurately and efficiently predict potential associations between miRNAs and diseases.

With the rapid development of artificial intelligence, numerous deep learning algorithms have been utilized to predict potential associations between miRNAs and diseases [14,15,16]. For example, AEMDA [17] applied a deep autoencoder (AE) to observe unconfirmed miRNA–disease interactions. Subsequently, VGAEMDA [18] applied a variational graph autoencoder to predict miRNA–disease associations. Specifically, VGAEMDA constructed miRNA-based and disease-based graphs, which were used by two graph autoencoders (GAEs) to generate miRNA–disease association scores. Then, the scores from these two trained networks were integrated to acquire the final predicted association scores for miRNA–disease pairs. GONNMDA [19] integrated similarity features from multiple data sources and applied noise reduction to obtain a refined and comprehensive similarity representation. Subsequently, they constructed heterogeneous graphs and employed a root–tree hierarchical alignment strategy, together with an ordered gating message-passing mechanism, which effectively addressed the challenges of data heterogeneity and over-smoothing. Finally, a multilayer perceptron (MLP) was used to generate the predicted associations. SFPred [20] first quantified the importance of one miRNA (or disease) node to another based on subgraphs formed by their neighbors. As graph encoding deepens within the miRNA–disease heterogeneous network, node features are progressively updated. To reflect this dynamic, an MLP-based strategy was introduced to estimate edge weights and construct an adaptive graph topology. Furthermore, a context-aware transformer was designed to capture contextual relationships between miRNA–disease node pairs. MAMFGAT [21] adopted a graph attention network (GAT) as the core framework for feature aggregation and integrated a multi-modal adaptive fusion module to extract representations from three interrelated networks: the miRNA–disease association network, the miRNA similarity network, and the disease similarity network. It leveraged adaptive learning and cross-modality contrastive learning to effectively fuse miRNA and disease embeddings. Additionally, a multi-modal residual fusion mechanism was introduced to mitigate the over-smoothing issue commonly encountered in GATs. Finally, an MLP was employed to predict potential miRNA–disease associations based on the learned embeddings.

Although recent deep learning-based methods have shown promising performance in miRNA–disease association prediction, several limitations remain. Firstly, many existing approaches mainly focus on nonlinear representations learned from similarity networks or graph structures, while the global linear structure contained in the miRNA–disease association matrix may not be sufficiently exploited [22,23]. Secondly, when heterogeneous representations are available, they are often directly concatenated or fused without explicitly modeling the consistency between different representation spaces. Thirdly, a simple combination of miRNA and disease features may not adequately capture interactions between the two entities. These limitations motivate the development of a framework that can jointly exploit complementary linear and nonlinear information, coordinate heterogeneous representations, and further model pair-level feature interactions [24,25].

To address these limitations, we developed SGMDA as a computational framework for miRNA–disease association prediction. SVD is used to capture global linear structural patterns from the association matrix, while a GAE learns nonlinear representations from integrated similarity graphs. Contrastive learning is subsequently introduced to improve the consistency between these complementary representation spaces rather than directly combining them. The aligned miRNA and disease representations are further processed by a highway network to model feature interactions before final classification. The main contributions of this study are summarized as follows:

- We developed a complementary representation-learning framework that jointly captures global linear structural information from the miRNA–disease association matrix and nonlinear information from integrated similarity graphs.
- We introduced a contrastive integration strategy to improve the consistency between heterogeneous linear and nonlinear representations, rather than directly combining the two feature spaces.
- We further modeled interactions between the integrated miRNA and disease representations through a highway network before association prediction.
- We conducted cross-validation experiments and case studies to evaluate the performance of SGMDA, and the results showed that SGMDA achieved competitive performance compared with the baseline methods.

## 2. Materials

### 2.1. Known miRNA–Disease Associations

In this paper, we utilized known miRNA–disease association information downloaded from the HMDD v3.2 database [26], which contained 12,446 verified associations between 591 diseases and 853 miRNAs. In order to facilitate calculation, the adjacency matrix $A\in R^{nd\times nm}$ was constructed to represent the miRNA–disease relationship, where nd and nm denote the number of diseases and miRNAs, respectively. If disease $d_{i}$ is verified to be associated with $m_{j}$, A(i,j)=1; otherwise, A(i,j)=0.

### 2.2. Disease Semantic Similarity

The directed acyclic graph (DAG) based on the MeSH descriptor [27] can be utilized to describe diseases. In this section, we applied the DAG(D)=(D,T(D),E(D)) to represent the DAG of disease *D*. T(D) denotes the nodes in the DAG that include *D* itself and its ancestor nodes. E(D) denotes the edges in the DAG that connect child nodes with parent nodes directly. The formulas to calculate the semantic contribution value of ancestor disease *d* and the semantic score of disease *D* are shown as follows:

(1) $$DV1\left(D\right)=\sum_{d\in T(D)}D_{D}1\left(d\right),$$

(2) $$\left\{\begin{array}{c} D_{D}1\left(d\right)=1,\;\mathrm{if}\;d=D\\ D_{D}1\left(d\right)=\max\left\{\Delta D_{D}1\left(d^{\prime }\right)|d^{\prime }\in \;\mathrm{children}\;\mathrm{of}\;d\right\},\;\mathrm{if}\;d\neq D, \end{array}\right.$$

where Δ is the semantic contribution factor that is equal to 0.5 in our paper, based on the previous literature [28].

In addition, the semantic similarity score between disease $d_{i}$ and disease $d_{j}$ can be calculated using the following formula:

(3) $$SS_{1}\left(d_{i},d_{j}\right)=\frac{\sum_{t\in T\left(d_{i}\right)\cap T\left(d_{j}\right)}\left(D_{d_{i}}1\left(t\right)+D_{d_{j}}1\left(t\right)\right)}{DV1\left(d_{i}\right)+DV1\left(d_{j}\right)}.$$

Diseases located at the same level of a DAG have the same semantic contribution value under the first scheme. However, specific diseases occur in only a few DAGs and should therefore be assigned higher semantic contributions [29].  Therefore, we introduced another way to calculate the semantic score of disease *D* and the contribution values of disease *d* as follows:

(4) $$DV2\left(D\right)=\sum_{d\in T(D)}D_{D}2\left(d\right),$$

(5) $$D_{D}2\left(d\right)=-\log\left(\frac{\;\mathrm{the}\;\mathrm{number}\;\mathrm{of}\;\mathrm{DAGs}\;\mathrm{including}\;d}{\;\mathrm{the}\;\mathrm{number}\;\mathrm{of}\;\mathrm{diseases}\;}\right).$$

Then, the semantic similarity score between diseases $d_{i}$ and $d_{j}$ can be calculated using the following formula:

(6) $$SS_{2}\left(d_{i},d_{j}\right)=\frac{\sum_{t\in T\left(d_{i}\right)\cap T\left(d_{j}\right)}\left(D_{d_{i}}2\left(t\right)+D_{d_{j}}2\left(t\right)\right)}{DV2\left(d_{i}\right)+DV2\left(d_{j}\right)}.$$

Finally, we integrated $SS_{1}$ and $SS_{2}$ to calculate the final disease semantic similarity $SD_{1}\left(d_{i},d_{j}\right)$ between diseases $d_{i}$ and $d_{j}$ as follows:

(7) $$SD_{1}\left(d_{i},d_{j}\right)=\frac{SS_{1}\left(d_{i},d_{j}\right)+SS_{2}\left(d_{i},d_{j}\right)}{2}.$$

### 2.3. miRNA Functional Similarity

Based on the assumption that miRNAs with similar functions tend to be associated with similar diseases, and vice versa, we calculated miRNA functional similarity according to the MISIM framework [30,31]. Specifically, the functional similarity between two miRNAs was estimated based on the disease semantic similarities defined in the previous section. For each disease associated with one miRNA, its similarity to the disease set associated with the other miRNA was determined by the maximum semantic similarity between this disease and the diseases in the corresponding set. The similarities were then aggregated in both directions between the two disease sets and normalized according to the numbers of associated diseases to obtain the functional similarity between the two miRNAs. For convenient calculation, we constructed the matrix $SM_{1}\in R^{nm\times nm}$ to represent the miRNA functional similarity information, where $SM_{1}\left(m_{i},m_{j}\right)$ denotes the functional similarity between miRNA $m_{i}$ and miRNA $m_{j}$.

### 2.4. Gaussian Interaction Profile Kernel Similarity

Based on the previous research, Gaussian interaction profile (GIP) kernel similarity could be applied to denote disease and miRNA similarity [32,33]. Therefore, we used vector $K(d_{i})$ to express the interaction profile of disease $d_{i}$ according to whether $d_{i}$ had a known connection with each miRNA or not. Then, we utilized the following equation to calculate GIP kernel similarity between disease $d_{i}$ and disease $d_{j}$:

(8) $$SD_{2}\left(d_{i},d_{j}\right)=\exp\left(-\gamma_{d}{∥K\left(d_{i}\right)-K\left(d_{j}\right)∥}^{2}\right),$$

where $\gamma_{d}$ is used to control kernel bandwidth and obtained by normalizing the initial bandwidth $\gamma_{d}^{\prime }$ to the average number of known interactions with miRNAs per disease as follows:

(9) $$\gamma_{d}=\gamma_{d}^{\prime }/\left(\frac{1}{nd}\sum_{i=1}^{nd}{∥K\left(d_{i}\right)∥}^{2}\right).$$

Similarly, we utilized the following equation to calculate GIP kernel similarity between miRNA $m_{i}$ and miRNA $m_{j}$:

(10) $$SM_{2}\left(m_{i},m_{j}\right)=\exp\left(-\gamma_{m}{∥K\left(m_{i}\right)-K\left(m_{j}\right)∥}^{2}\right),$$

where $\gamma_{m}$ is also used to control kernel bandwidth and obtained by the following formula:

(11) $$\gamma_{m}=\gamma_{m}^{\prime }/\left(\frac{1}{nm}\sum_{i=1}^{nm}{∥K\left(m_{i}\right)∥}^{2}\right).$$

### 2.5. Integrating Similarity for miRNAs and Diseases

Considering that we cannot acquire miRNA functional similarity for all miRNAs, $SM_{1}$ and $SM_{2}$ are integrated to form the miRNA similarity matrix SM with complete data as follows:

(12) $$SM\left(m_{i},m_{j}\right)=\left\{\begin{array}{cc} SM_{1}\left(m_{i},m_{j}\right), & \mathrm{if}\;SM_{1}\left(m_{i},m_{j}\right)\neq 0\\ SM_{2}\left(m_{i},m_{j}\right), & \mathrm{otherwise}. \end{array}\right.$$

Similarly, $SD_{1}$ and $SD_{2}$ are integrated to construct the disease similarity matrix SD with rich data as follows:

(13) $$SD\left(d_{i},d_{j}\right)=\left\{\begin{array}{cc} SD_{1}\left(d_{i},d_{j}\right), & \mathrm{if}\;SD_{1}\left(d_{i},d_{j}\right)\neq 0\\ SD_{2}\left(d_{i},d_{j}\right), & \mathrm{otherwise}. \end{array}\right.$$

## 3. Method

**Figure 1:**
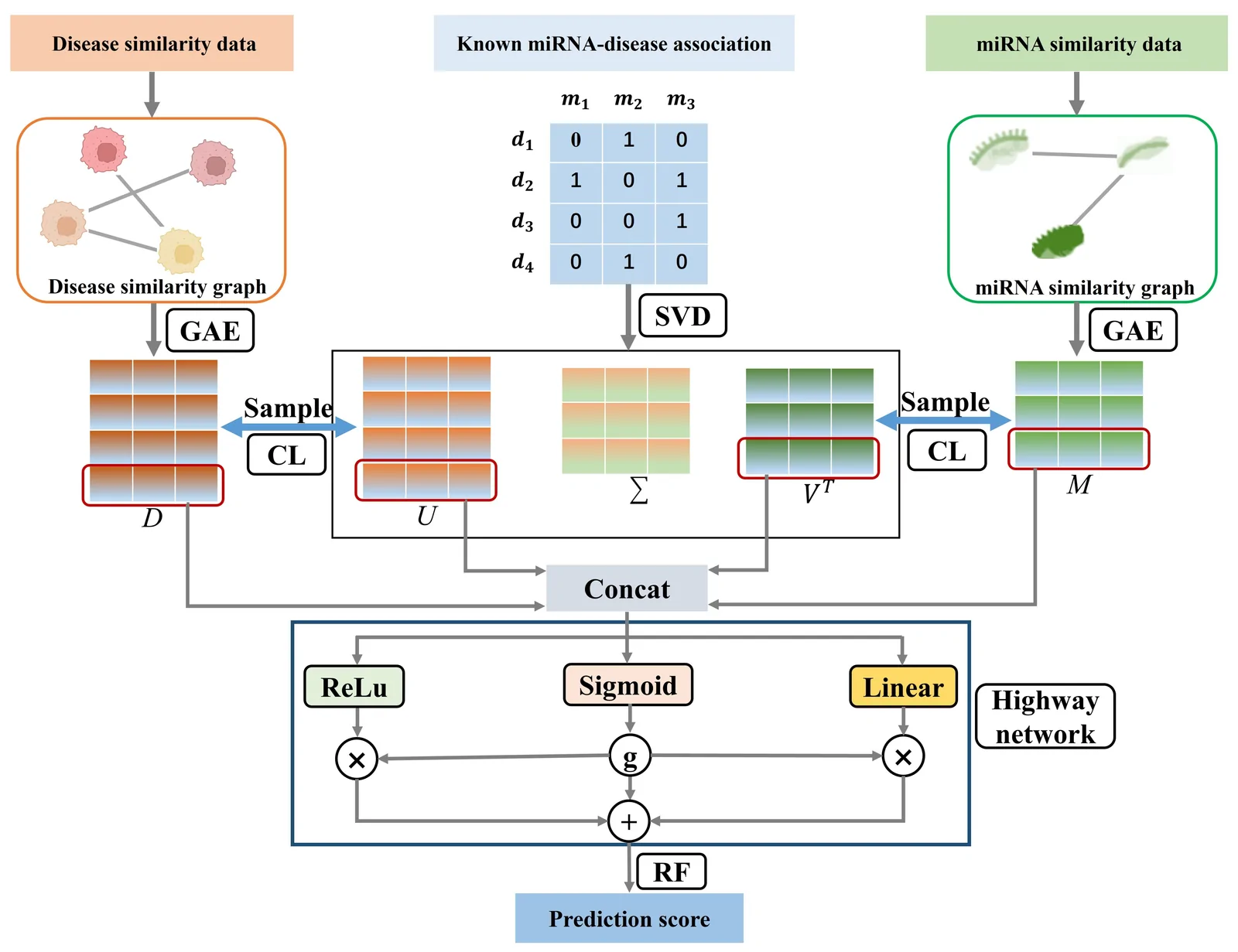
The SGMDA flow chart. Singular value decomposition (SVD) and a graph autoencoder (GAE) are employed to extract linear and nonlinear features of miRNAs and diseases based on the association matrix and similarity graph, respectively. Contrastive learning (CL) is then applied to refine these features by enhancing their consistency and discriminability. A highway network is utilized to learn interactions among the concatenated features and adjust the feature representation accordingly. Finally, a random forest (RF) classifier is used to perform prediction based on the adjusted feature.

In this paper, we propose a novel prediction method named SGMDA, which combines the advantages of singular value decomposition and deep learning to predict possible miRNA–disease associations. The SGMDA flow chart is presented in Figure 1.

### 3.1. Extracting Linear Features with Singular Value Decomposition

Matrix factorization algorithms have been widely used in recommendation systems, and SVD is regarded as one of the most widely used matrix factorization algorithms [34]. Hence, we used the SVD algorithm in our model to extract linear features of miRNAs and diseases.

According to the theory of the SVD algorithm, the matrix *A* can be factorized into *U*, Σ and $V^{T}$ using the following formula:

(14) $$A\approx U\Sigma V^{T},$$

where $U\in R^{nd\times k}$ and $V^{T}\in R^{k\times nm}$ are real matrices, and $\Sigma \in R^{k\times k}$ is a diagonal matrix in which each value on the diagonal denotes the significance of its mapped attribute. In this study, we regard the vectors $U_{i}$ and $V_{j}^{T}$ obtained from singular value decomposition (SVD) as the linear feature representations of disease $d_{i}$ and miRNA $m_{j}$, respectively. This is grounded in the theoretical foundation of SVD, which decomposes the original miRNA–disease association matrix into the product of three matrices, capturing the most significant directions of variation in the data. Specifically, matrices *U* and *V* serve as orthogonal bases that project the original high-dimensional data into a lower-dimensional latent space through a linear transformation. These projections encode the most informative linear patterns within the data and reflect the primary structural components of the original matrix. Unlike nonlinear approaches such as multilayer perceptron or deep neural networks, SVD is entirely based on linear decomposition, and thus its resulting embeddings are inherently linear. Therefore, $U_{i}$ and $V_{j}^{T}$ can be interpreted as compressed linear embeddings that preserve essential information from the original matrix.

### 3.2. Extracting Nonlinear Features with Graph Autoencoder

To extract structure-aware nonlinear features of diseases and miRNAs, we constructed two separate similarity graphs: a disease similarity graph and a miRNA similarity graph. In each graph, nodes represent diseases or miRNAs, and edges encode their pairwise similarities. Only similarity values greater than 0.5 were retained to form edges, ensuring that only strong relationships were incorporated into the graph structure. We then applied graph autoencoders to these graphs to obtain compact and informative low-dimensional embeddings for each node.

The GAE model consists of two parts: a graph encoder and a decoder [35]. The encoder adopts graph convolutional networks (GCNs) to project node features into a latent embedding space while preserving both attribute and topological information. To illustrate, we take the operations on the disease similarity graph as an example. The adjacency matrix $A^{s}$ is built by connecting nodes with similarity scores greater than 0.5. The feature matrix *X* is a one-hot encoding, where each node is represented by a unique identity vector. The encoding process is formulated as

(15) $$Z=\mathrm{GCN}\left(A^{s},X\right)=\sigma \left(\tilde{A^{s}}XW\right),$$

where *W* is a trainable weight matrix, $\tilde{A^{s}}$ is the symmetrically normalized adjacency matrix with self-loops, and σ(·) is the ReLU activation function. The output embedding matrix is denoted by $Z\in R^{n\times d_{g}}$, where *n* is the number of disease nodes and $d_{g}$ denotes the dimension of the nonlinear representation learned by the GAE.

The decoder aims to reconstruct the similarity structure of the graph by computing the inner product between node embeddings:

(16) $${\hat{A}}_{ij}^{s}=\sigma_{d}\left(z_{i}^{⊤}z_{j}\right),$$

where $z_{i}$ and $z_{j}$ are the learned embeddings of nodes $d_{i}$ and $d_{j}$, respectively, and $\sigma_{d}\left(\cdot \right)$ is a Sigmoid activation function to ensure outputs lie in [0, 1].

The training objective minimizes the reconstruction loss between the original similarity-graph adjacency matrix $A^{s}$ and the reconstructed matrix ${\hat{A}}^{s}$:

(17) $$L_{DG}=-\sum_{i,j}\left[A_{ij}^{s}\log{\hat{A}}_{ij}^{s}+\left(1-A_{ij}^{s}\right)\log\left(1-{\hat{A}}_{ij}^{s}\right)\right],$$

which is a binary cross-entropy loss commonly used in GAE models.

By applying the above GAE model to the disease similarity graph, we obtain the low-dimensional latent representation $D_{i}$ for disease $d_{i}$, with the corresponding reconstruction loss denoted by $L_{DG}$. Similarly, applying GAE to the miRNA similarity graph yields the representation $M_{j}$ for each miRNA $m_{j}$, and the corresponding reconstruction loss is denoted by $L_{MG}$. These embeddings, $D_{i}$ and $M_{j}$, capture both feature-level and topology-aware information for downstream prediction tasks. Accordingly, the overall GAE reconstruction loss is defined as $L_{\mathrm{rec}}=L_{DG}+L_{MG}$.

### 3.3. Contrastive Learning Optimization

Linear features typically preserve global structural information, with features extracted via SVD capturing overall correlation patterns. In contrast, nonlinear features are better suited for modeling complex, localized, and nonlinear relationships. While both types of features offer unique advantages, simple concatenation often fails to fully exploit their complementary strengths. Contrastive learning (CL) enhances both the consistency and distinctiveness of these representations in the embedding space by pulling together positive samples and pushing apart negative ones, thereby enabling more effective feature fusion.

In this section, we utilized a CL strategy to minimize the distance between the representations of the same entity under linear and nonlinear feature spaces, while maximizing the distances between representations of different entities. Specifically, for disease $d_{i}$, we treated its representation $U_{i}$ derived from the linear view as the anchor and its representation $D_{i}$ from the nonlinear view as the positive sample. Representations of all other diseases were regarded as negative samples. Through this contrastive mechanism, the model effectively pulled closer the feature representations of the same disease under different encodings, while pushing apart those of different diseases. An identical strategy was applied to miRNA $m_{j}$.

This approach facilitated the alignment of linear and nonlinear features, alleviated representation homogeneity, and significantly enhanced the consistency and discriminability of the learned embeddings. The contrastive loss for diseases was defined as

(18) $$\begin{array}{c} L_{DC}=-\sum_{i\in I_{d}}\log\left(\frac{\exp\left(\mathrm{sim}(U_{i},D_{i})/T\right)}{\sum_{j\in I_{d}}\sum_{s\in \{U_{j},D_{j}\}}\exp\left(\mathrm{sim}(U_{i},s)/T\right)}\right), \end{array}$$

where $I_{d}$ denotes the index set of all disease samples, sim(x,y) represents the cosine similarity, and *T* is the temperature hyperparameter. Here, $s\in \{U_{j},D_{j}\}$ denotes the linear and nonlinear representations included in the denominator, while the representations of diseases other than the anchor disease are treated as negative samples. Similarly, the contrastive loss for miRNAs, denoted by $L_{MC}$, is formulated in the same manner by treating the linear and nonlinear representations of the same miRNA as a positive pair and the representations of other miRNAs as negative samples. Accordingly, the overall CL loss is defined as $L_{\mathrm{CL}}=L_{DC}+L_{MC}$.

### 3.4. Learning Feature Interactions Through Highway Network

The SVD-derived linear representation and the GAE-derived nonlinear representation of disease $d_{i}$ are concatenated to construct its fused representation. The corresponding linear and nonlinear representations of miRNA $m_{j}$ are concatenated in the same manner. The resulting disease and miRNA representations are then combined to construct a pair-level feature $F_{ij}$ for the miRNA–disease pair $\left(m_{j},d_{i}\right)$. Direct concatenation combines miRNA and disease features without explicitly learning interactions among their components. We therefore introduce a highway network to process the pair-level features through gated transformations.

The highway network combines nonlinear and linear transformations of the input feature:

(19) $$F_{b,ij}=g_{ij}⊙t\left(F_{ij}\right)+\left(1-g_{ij}\right)⊙q\left(F_{ij}\right),$$

where $F_{b,ij}$ denotes the resulting pair representation, $t(F_{ij})$ is a learnable nonlinear transformation with ReLU activation, and $q(F_{ij})$ is a learnable linear transformation. Both transformations preserve the dimensionality of $F_{ij}$ to permit element-wise combination. The input-dependent gate is defined as

(20) $$g_{ij}=\sigma \left(F_{ij}\right),$$

where σ(·) denotes the sigmoid function. This gate introduces no additional trainable parameters. The complementary weights $g_{ij}$ and $\left(1-g_{ij}\right)$ control the contributions of the nonlinear and linear branches, respectively.

To train the highway network without introducing an additional classification head, we apply a supervised contrastive objective directly to its output representations. For a training mini-batch B, let $h_{a}=F_{b,i_{a}j_{a}}$ denote the highway representation of the *a*th training pair $\left(m_{j_{a}},d_{i_{a}}\right)$, and let $y_{a}\in \left\{0,1\right\}$ denote its training label. Label 1 indicates a known training association, whereas label 0 indicates an unlabeled pair sampled as a negative training instance. For computing the supervised contrastive objective, the highway representation is normalized as

(21) $$z_{a}=\frac{h_{a}}{\max({∥h_{a}∥}_{2},\epsilon )},$$

where ϵ>0 ensures numerical stability. For each anchor *a*, the set of other training samples with the same label is defined as

(22) $$S\left(a\right)=\{p\in B\setminus \left\{a\right\}:y_{p}=y_{a}\}.$$

Each mini-batch contains at least two distinct samples from each class, ensuring that S(a) is nonempty. The supervised contrastive loss is

(23) $$L_{\mathrm{HW}}=-\frac{1}{\left|B\right|}\sum_{a\in B}\frac{1}{\left|S(a)\right|}\sum_{p\in S(a)}\log\frac{\exp(z_{a}^{⊤}z_{p}/\tau_{\mathrm{HW}})}{\sum_{b\in B\setminus \{a\}}\exp\left(z_{a}^{⊤}z_{b}/\tau_{\mathrm{HW}}\right)},$$

where $\tau_{\mathrm{HW}}>0$ denotes the temperature parameter of the pair-level supervised contrastive objective. This objective encourages training pairs with the same training label to have more similar normalized representations relative to pairs with different labels. The same-label samples in S(a) are defined for contrastive learning and should not be confused with experimentally supported positive associations. Unlike the node-level contrastive objective used to align linear and nonlinear representations, $L_{\mathrm{HW}}$ provides label supervision directly to the pair-level highway representations.

### 3.5. Making Predictions by Random Forest

Random forest (RF) is a widely used machine learning algorithm that can be implemented to perform not only classification tasks but also regression tasks [36]. In particular, RF contains bootstrap and random sample splitting methods. In bootstrap-based random resampling with replacement, many decision trees are trained and integrated into a forest to predict the category or target variable values of unknown samples. In addition, unlike the common decision tree algorithm, a set number of input variables are randomly selected to conduct a split node at each node, and no pruning step is implemented in the process of training decision trees in the random forest. Hence, the RF can achieve robust and outstanding performance.

In our study, the learned miRNA–disease pair representations were obtained after the neural representation-learning stages were fixed and used as input features for the random forest classifier. Each positive miRNA–disease pair was labeled as 1, and each sampled negative pair was labeled as 0. The random forest was then trained independently using these labeled training samples and did not participate in gradient-based optimization. After training, the classifier assigned a prediction score to each candidate miRNA–disease pair, with a higher score indicating a greater likelihood of association between the miRNA and the disease.

### 3.6. Model Training and Optimization

The trainable neural components of SGMDA are jointly optimized using Adam under a unified objective. This objective jointly considers graph reconstruction, node-level contrastive alignment, and pair-level supervised contrastive learning. The overall training objective is defined as

(24) $$L_{\mathrm{total}}=L_{\mathrm{rec}}+L_{\mathrm{CL}}+L_{\mathrm{HW}},$$

where $L_{\mathrm{rec}}$ denotes the GAE reconstruction loss, $L_{\mathrm{CL}}$ denotes the node-level contrastive loss, and $L_{\mathrm{HW}}$ denotes the supervised contrastive loss for the highway network.

During optimization, the SVD-derived linear representations remain fixed and are not updated according to gradient descent. After the trainable neural components are optimized, their parameters are fixed, and the learned pair representations are subsequently used to train the random forest classifier independently. The random forest does not participate in the gradient-based optimization.

## 4. Results

### 4.1. Experiment Setting

SGMDA was implemented in Python (version 3.14.6) using PyTorch (version 2.1.0), an open-source machine learning framework. All experiments were conducted on an Ubuntu system equipped with two NVIDIA A100 GPUs. The source code is available at https://github.com/lilei0203/SGMDA (accessed on 1 October 2026).

To evaluate the prediction ability of SGMDA, we compared SGMDA with five baseline models named AEMDA [17], VGAEMDA [18], GONNMDA [19], SFPred [20], and MAMFGAT [21] under 5-fold cross-validation (5-CV), global leave-one-out cross-validation (LOOCV), miRNA-wise cross-validation (M-CV), and disease-wise cross-validation (D-CV).

In 5-CV, we considered the labeled associations in the HMDD v3.2 database as positive samples and randomly selected an equal number of unlabeled miRNA–disease pairs as negative samples, resulting in a balanced dataset. To reduce the potential influence of a particular negative-sample selection, the negative samples were independently resampled in each run, and the experiments were repeated 20 times. Within each run, the same sampled negative pairs were used for SGMDA and all competing methods to ensure a fair comparison. The resulting dataset was randomly divided into five folds, with each fold serving as the test set in turn and the remaining four folds used for training. For each fold of 5-CV, the held-out associations were first removed from the miRNA–disease association matrix. The linear feature representations derived by SVD, the GIP-based similarity measures, and the association-dependent miRNA functional similarities were then recomputed using only the remaining training associations. The held-out associations were used exclusively for evaluation. Algorithm 1 summarizes the 5-CV evaluation procedure of SGMDA. In global LOOCV, each known miRNA–disease association in the HMDD v3.2 dataset was individually held out as a positive test instance, while the remaining known associations were used for training. In each iteration, the held-out association was first removed from the association matrix, and all association-derived features and representations were then reconstructed using only the remaining training associations, following the same procedure as in 5-CV. For model training, an equal number of unobserved miRNA–disease pairs were randomly sampled as negative training instances, resulting in a 1:1 ratio of positive to sampled negative training instances. During testing, the held-out association was treated as the positive test instance, while all unobserved miRNA–disease pairs were used as candidate negative samples. The trained model generated prediction scores for the held-out association and the candidate pairs, which were ranked in descending order according to their prediction scores. The ROC and Precision–Recall curves were constructed by varying the score threshold, from which AUC and AUPR were calculated. For ACC, Precision, Recall, and F1, the classification threshold was determined using only the training data and was subsequently fixed when evaluating the held-out test candidates. This process was repeated until every known association had been held out exactly once. The entire global LOOCV experiment was repeated 20 times with independently sampled negative training instances. Algorithm 2 summarizes the global LOOCV evaluation procedure of SGMDA. The reported performance metrics were summarized as mean ± standard deviation over 20 independent runs.

To further evaluate the generalization ability of SGMDA to entities with no observed associations in the training set, we additionally conducted miRNA-wise cross-validation (M-CV) and disease-wise cross-validation (D-CV). In M-CV, all miRNAs were randomly divided into five disjoint folds. In each fold, all known associations involving the miRNAs in the held-out fold were removed from the training set and used exclusively for evaluation, so that the test miRNAs had no known miRNA–disease associations available during model training. All association-derived information was reconstructed using only the remaining training associations. For evaluation, the held-out positive associations were combined with an equal number of randomly sampled unobserved pairs involving the same test miRNAs, resulting in a balanced test set. The unobserved pairs were independently resampled in each run, and the experiments were repeated 20 times. D-CV followed the same procedure, except that diseases rather than miRNAs were divided into five folds and all associations involving the held-out diseases were excluded from training. This setting was used to evaluate the ability of SGMDA to generalize to previously unseen diseases.
**Algorithm 1** 5-CV evaluation of SGMDA**Require:** miRNA–disease association matrix *A*; disease semantic similarity information; number of runs R=20**Ensure:** Held-out prediction scores and labels for each run  1:$P\leftarrow \{\left(i,j\right)|A_{ij}=1\}$  2:$U\leftarrow \{\left(i,j\right)|A_{ij}=0\}$  3:**for** r=1,…,R **do**  4:     Randomly sample $N_{r}\subseteq U$ such that $|N_{r}\left|=|P\right|$  5:     Construct $D_{r}=P\cup N_{r}$, assigning labels 1 and 0, respectively  6:     Randomly divide $D_{r}$ into five disjoint folds $F_{r,1},\ldots ,F_{r,5}$  7:     **for** k=1,…,5 **do**  8:         $D_{\mathrm{test}}\leftarrow F_{r,k}$; $D_{\mathrm{train}}\leftarrow D_{r}\setminus F_{r,k}$  9:         Construct $A^{\mathrm{train}}$ with entries equal to 1 only for positive training associations and 0 otherwise10:         Extract linear feature representations by SVD from $A^{\mathrm{train}}$11:         Recompute GIP-based similarities from $A^{\mathrm{train}}$12:         Recompute miRNA functional similarities using the training associations and disease semantic similarities13:         Construct the integrated miRNA and disease similarity graphs for the current fold14:         Jointly optimize the trainable neural components using $L_{\mathrm{total}}$ on the current-fold training data, while keeping the SVD-derived representations fixed15:         Extract the learned training-pair representations using the optimized neural components16:         Train the RF classifier using the training-pair representations and training labels17:         Generate scores for the held-out test pairs using the current-fold representation model and trained classifier18:         Store the held-out scores and their corresponding labels for evaluation19:    **end for**20:**end for**

**Algorithm 2** Global LOOCV evaluation of SGMDA**Require:** miRNA–disease association matrix *A*; disease semantic similarity information; number of runs R=20**Ensure:** Held-out prediction scores, rankings, and labels for each run
  1:

$P\leftarrow \{\left(i,j\right)|A_{ij}=1\}$

  2:

$U\leftarrow \{\left(i,j\right)|A_{ij}=0\}$

  3:**for** r=1,…,R **do**  4:      **for all** p∈P **do**  5:             Treat *p* as the held-out positive test association  6:             $P_{\mathrm{train}}\leftarrow P\setminus \left\{p\right\}$  7:             Randomly sample $N_{r,p}\subseteq U$ such that $|N_{r,p}\left|=\right|P_{\mathrm{train}}|$  8:             Construct $D_{\mathrm{train}}=P_{\mathrm{train}}\cup N_{r,p}$, assigning labels 1 and 0, respectively  9:             Construct $A^{\mathrm{train}}$ with entries equal to 1 only for positive training associations and 0 otherwise10:             Extract linear feature representations by SVD from $A^{\mathrm{train}}$11:             Recompute GIP-based similarities from $A^{\mathrm{train}}$12:             Recompute miRNA functional similarities using the training associations and disease semantic similarities13:             Construct the integrated miRNA and disease similarity graphs for the current iteration14:             Jointly optimize the trainable neural components using $L_{\mathrm{total}}$ on $D_{\mathrm{train}}$, while keeping the SVD-derived representations fixed15:             Extract the learned training-pair representations using the optimized neural components16:             Train the RF classifier using the training-pair representations and training labels17:             Determine the classification threshold using the training data only18:             Generate scores for the held-out association *p* and all unobserved candidate pairs in U19:             Rank the held-out association and candidate pairs in descending order according to their prediction scores20:             Store the prediction scores, rankings, and  threshold-based labels for evaluation21:      **end for**22:
**end for**



### 4.2. Performance Comparison

This section showcases the efficacy of our proposed model by presenting the prediction performances. We compare SGMDA with baseline methods under 5-CV and report the results of these methods in Table 1. The performance values reported for SGMDA were an AUC of 0.94 ± 0.01, AUPR of 0.93 ± 0.01, Accuracy of 0.94 ± 0.02, Precision of 0.92 ± 0.01, Recall of 0.91 ± 0.02, and F1 score of 0.91 ± 0.01. The statistical significance of AUC differences is also presented in Table 1. Table 2 presents the prediction performance of SGMDA and baseline models under global LOOCV. The results demonstrate that SGMDA consistently outperforms all other models. Specifically, AEMDA and VGAEMDA rely solely on autoencoders and variational graph autoencoders to extract nonlinear features from relatively simple data sources. In contrast, GONNMDA, SFPred, and MAMFGAT learn nonlinear representations from richer and more diverse data, leading to their superior performance compared to the former two models. This comparison highlights the importance of feature richness in enhancing model performance. Notably, SGMDA integrates both linear features and nonlinear representations derived from comprehensive and heterogeneous data sources, effectively capturing both structural relationships and intricate nonlinear patterns. This rich feature integration enables the model to leverage complementary information across different feature spaces, thereby enhancing its expressive power and predictive performance.

Meanwhile, although SFPred incorporates graph contextual modeling and attention mechanisms, it lacks an explicit design for selectively modeling feature interactions. AEMDA and VGAEMDA rely solely on MLPs to process concatenated features, which tends to overlook complex nonlinear interactions. GONNMDA places greater emphasis on graph structure construction and processing, but its capability to model detailed feature interactions remains limited. In contrast, SGMDA leverages a highway network to capture the deep interactions between miRNA and disease features. This architecture facilitates the selective transmission and integration of information across layers, thereby improving the representation capacity and predictive performance of SGMDA.

The generalization ability of SGMDA was further evaluated under the more challenging entity-wise cross-validation settings. Under M-CV, as shown in Table 3, SGMDA achieved AUC, AUPR, ACC, Precision, Recall, and F1 scores of 0.92±0.01, 0.91±0.02, 0.92±0.02, 0.90±0.01, 0.90±0.01, and 0.90±0.01, respectively. Compared with the standard pair-wise 5-CV setting, the AUC and AUPR decreased by approximately 0.02 and 0.02, respectively, reflecting the increased difficulty of predicting associations for miRNAs with no observed associations in the training set. Nevertheless, SGMDA still achieved the highest AUC and AUPR among the compared methods, exceeding the strongest baseline by 0.01 in both metrics. A similar but more pronounced performance decline was observed under D-CV. As shown in Table 4, SGMDA obtained AUC, AUPR, ACC, Precision, Recall, and F1 scores of 0.90±0.01, 0.89±0.02, 0.90±0.02, 0.89±0.01, 0.88±0.01, and 0.88±0.01, respectively. Relative to pair-wise 5-CV, the AUC and AUPR decreased by approximately 0.04 and 0.05, respectively, indicating that generalization to previously unseen diseases is more challenging than association completion among already observed entities. Despite this decline, SGMDA outperformed the strongest baseline by 0.02 in both AUC and AUPR. Overall, these results indicate that SGMDA maintains effective predictive performance under more challenging cold-start conditions involving previously unseen miRNAs and diseases.

To statistically assess the performance differences between SGMDA and the baseline methods, paired *t*-tests were performed on the AUC scores obtained from 20 independent runs. Within each run, all methods used the same data partitions and sampled negative pairs. The normality of the paired AUC differences was examined before statistical testing, and the resulting *p*-values were adjusted using the Holm method for multiple comparisons. The significance level was set at 0.05. The statistical analysis showed that SGMDA achieved significantly higher AUC values than the compared methods under the evaluated conditions. Thus, SGMDA is demonstrated to have sufficient ability to infer potential miRNA–disease associations.

### 4.3. Parameter Sensitivity Analysis

The architecture and performance of SGMDA are influenced by several parameter settings, including the similarity threshold used to construct the disease and miRNA similarity graphs, the dimensions of the linear representations extracted by SVD and the nonlinear representations learned by GAE, the temperature parameter *T* for node-level contrastive learning, the temperature parameter $\tau_{\mathrm{HW}}$ for pair-level supervised contrastive learning, and the learning rate, dropout, training epochs, and the main parameters of the RF classifier. Given that exhaustively evaluating all possible parameter combinations is computationally impractical, we adopted a grid-like search strategy for parameter tuning. For each fold of the 5-CV, the training portion was further divided into an inner training subset and a validation subset for parameter selection. Candidate parameter settings were selected according to the validation performance, while the corresponding test fold was kept completely unseen during this process. The final parameter settings are summarized in Table 5. The effects of different parameter settings on model performance were generally consistent across different cross-validation settings. Among these parameters, the similarity threshold directly determines the connectivity of the similarity graphs, while the dimensions of the linear and nonlinear representations affect the representation capacity of the model. Therefore, we further analyzed the sensitivity of SGMDA to these key parameters.

**Figure 2:**
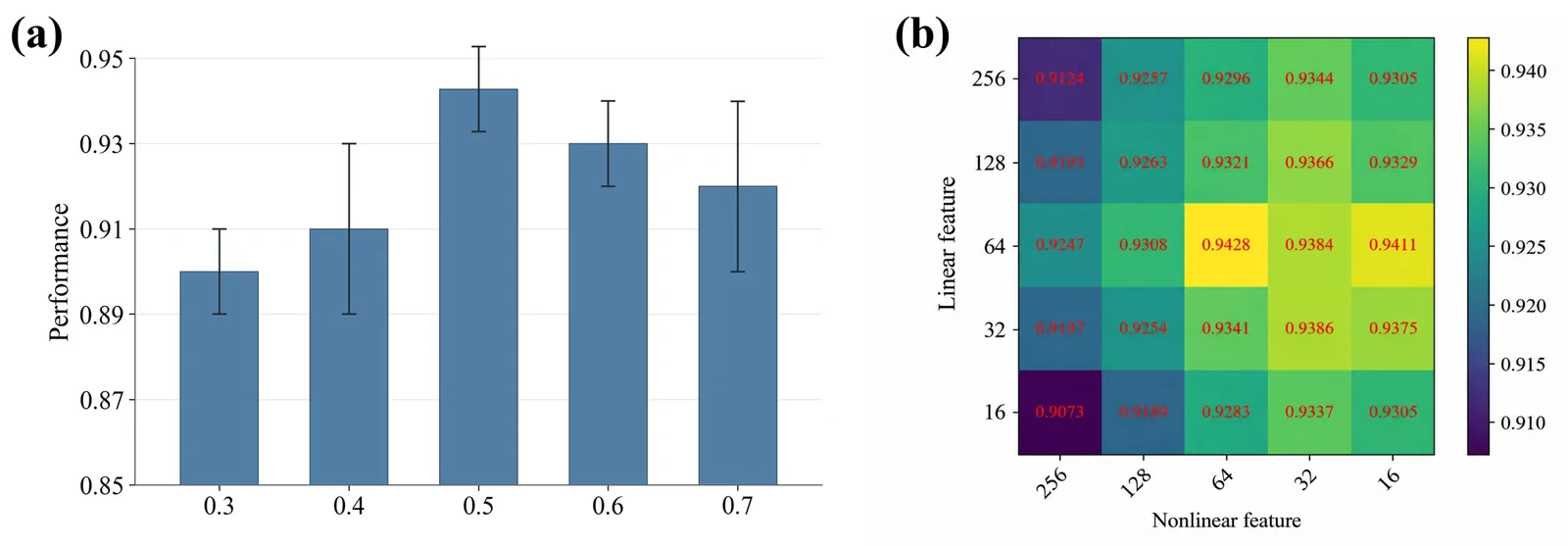
Sensitivity analysis of key parameters in SGMDA. (**a**) Effect of the similarity threshold on model performance. (**b**) Effect of different combinations of linear and nonlinear representation dimensions on model performance.

To assess the influence of the key parameters, we analyzed the similarity threshold used for graph construction and the dimensions of the linear and nonlinear representations. The similarity threshold determines the connectivity of the disease and miRNA similarity graphs: a small value may retain weak or noisy relationships, whereas a large value may lead to overly sparse graphs. Therefore, the threshold was varied over {0.3,0.4,0.5,0.6,0.7} while keeping the other settings fixed. For each threshold value, 20 independent validation runs were performed, and the distribution of the resulting AUC values is presented in Figure 2a. The validation performance first increased and then decreased as the threshold increased, with the best overall performance obtained at 0.5. Therefore, the similarity threshold was set to 0.5 in the subsequent experiments. We further evaluated the dimensions of the SVD-derived linear representations and GAE-derived nonlinear representations. Both dimensions were varied over {16,32,64,128,256}, and different combinations were evaluated using validation AUC. When the two dimensions were different, zero-padding was applied before contrastive learning. Each dimensional combination was evaluated over 20 independent validation runs, and the mean AUC of the 20 runs was used to construct Figure 2b. The highest mean validation AUC was obtained when both the linear and nonlinear representation dimensions were set to 64. Accordingly, both dimensions were set to 64 in the subsequent experiments.

### 4.4. Ablation Study

To evaluate the impact of linear and nonlinear features on model performance, as well as to assess the effectiveness of modules SVD, GAE, CL, and highway network, we conducted ablation experiments on the HMDD v3.2 dataset. The specific configurations of the ablation models are detailed as follows.

- SGMDA-LF discards linear features and trains the model using only nonlinear features.
- SGMDA-NF discards nonlinear features and trains the model using only linear features.
- SGMDA-SVD replaces SVD with non-negative matrix factorization to extract linear features.
- SGMDA-AE uses a GCN instead of a GAE to extract nonlinear features.
- SGMDA-CL no longer uses CL to optimize the linear and nonlinear features. Instead, it directly concatenates the features extracted by SVD and the GAE and feeds them into the highway network.
- SGMDA-HN no longer utilizes the highway network to learn feature interactions. Instead, it directly feeds the concatenated feature representations of miRNA–disease pairs into the RF for prediction.

As shown in Table 6, all ablation models exhibit a decline in performance compared to SGMDA under 5-CV. Removing nonlinear features led to a larger performance decrease than removing linear features. The model achieved its best performance when both linear and nonlinear features were used. The effectiveness of SVD and GAE as specialized feature extractors is confirmed, as their replacements lead to noticeable performance drops. Additionally, SGMDA-CL lacks the capability to effectively align and integrate complementary information across different feature spaces, which leads to decreased consistency and discriminability in the learned features. The straightforward feature fusion in SGMDA-HN overlooks the deep interactions between features, limiting the capacity of the model to capture complex nonlinear dependencies, which leads to a drop in predictive accuracy. Together, these components synergistically improve the predictive power of the model, supporting their complementary contributions to the reported results.

### 4.5. Case Study

To further evaluate the prediction performance of SGMDA, we selected two representative human diseases, lung neoplasms and prostate neoplasms, for case studies. All verified miRNA–disease associations in HMDD v3.2 were used to train the model. For each target disease, miRNAs with associations already recorded in HMDD v3.2 were excluded from the candidate set. The remaining unobserved miRNA–disease pairs were ranked according to their predicted association scores, and the top 50 candidates were selected for further validation using the dbDEMC v2.0 [37] and miR2Disease [38] databases.

Lung neoplasms are regarded as one of the most common diseases in males, and they cause great harm to men’s health. In recent years, several miRNAs associated with lung neoplasms have been observed in biological experiments [39]. For example, miR-98 and miR-93 have been shown to be associated with tumorigenesis in lung cancer [40]. After we ranked the candidate samples based on the predicted association scores, 48 of the top 50 lung neoplasm-related miRNAs were confirmed by the dbDEMC v2.0 and miR2Disease databases (Table 7). Notably, some highly ranked candidates that were not confirmed by dbDEMC v2.0 or miR2Disease have received additional support. For example, hsa-miR-769 iss not supported by either database in the current case study. Published experimental evidence indicates that miR-769-5p participates in the regulation of non-small cell lung cancer through the circ_0001955/miR-769-5p/EGFR axis, and the regulatory relationship between miR-769-5p and EGFR is experimentally confirmed [41]. These findings provide additional biological support for the predicted association between miR-769 and lung neoplasms, although further studies are still required to clarify its precise functional role in disease progression.

Prostate neoplasms are a disease with high incidence in the male population, and have been observed to be associated with several miRNAs in clinical experiments [42]. For example, the miR-183 expression is distinctly higher in prostate cancer cells and tissues, compared with that in corresponding normal prostate cells and tissues. Inhibition of miR-183 expression has shown potential antitumor effects in experimental models [43]. After we ranked the candidate samples based on the predicted association scores, 47 of the top 50 prostate neoplasm-related miRNAs are supported by at least one of dbDEMC v2.0 and miR2Disease databases (Table 8). Similar biological evidence was found for several highly ranked prostate-neoplasm candidates that were not supported by dbDEMC v2.0 or miR2Disease. For hsa-miR-122, subsequent experimental evidence showed that miR-122 suppresses the proliferation, migration, invasion, and tumorigenic potential of prostate cancer cells by targeting δ-catenin and modulating downstream factors including c-Myc, cyclin D1, Snail, and VEGF [44]. For hsa-miR-92a, Zhang et al. [45] reported that miR-92a regulates the growth, migration, and invasion of prostate cancer cells through SERTAD3 and p53-related signaling. Another study further showed that miR-92a is involved in prostate cancer cell proliferation, migration, invasion, and apoptosis through regulation of the PTEN/Akt signaling pathway [46]. These independent studies provide biological plausibility for several high-ranking predictions generated by SGMDA and suggest that these candidates warrant further experimental investigation.

To provide a more rigorous evaluation of the ability of SGMDA to identify previously unknown miRNA–disease associations, we further conducted a temporal validation experiment using different releases of HMDD. Specifically, the model was trained using only the miRNA–disease associations recorded in HMDD v2.0 [47], and all association-dependent inputs were constructed exclusively from this earlier release. Following the harmonization of miRNA and disease identifiers across the two releases, the candidate space was restricted to unrecorded associations between miRNAs and diseases already present in HMDD v2.0. Associations involving entities newly introduced into HMDD v3.2 were excluded from this analysis. After model training, all candidate miRNA–disease pairs that were not recorded in HMDD v2.0 were assigned prediction scores and ranked accordingly. Associations that were absent from HMDD v2.0 but subsequently included in HMDD v3.2 were regarded as newly added associations, and the ability of SGMDA to recover these associations among the top-ranked candidates was evaluated. Specifically, we evaluated the recovery of newly recorded HMDD v3.2 associations among the top 100, top 500, and top 1000 globally ranked candidate pairs, and reported the corresponding Precision@K values to quantify the ability of SGMDA to prioritize associations that were subsequently included in the later HMDD release. As shown in Table 9, a large proportion of the highest-ranked predictions generated from HMDD v2.0 were subsequently recorded in HMDD v3.2, providing retrospective support for the ability of SGMDA to prioritize potentially novel miRNA–disease associations.

The case studies provide additional database-supported evidence that SGMDA can prioritize potentially relevant disease-associated miRNAs. The database-supported predictions and temporal analysis should be interpreted in light of possible overlap in curated evidence and do not replace independent experimental validation.

## 5. Discussion

The experimental results suggest that the performance of SGMDA benefits from the complementary modeling of different types of information. Compared with methods that mainly rely on a single representation space, SGMDA combines the global linear structure extracted from the miRNA–disease association matrix with nonlinear representations learned from integrated similarity graphs. The contrastive learning module further encourages consistency between these heterogeneous representations, while the highway network models interactions between miRNA and disease features before classification. The ablation results also indicate that these components make complementary contributions to the final prediction performance. These findings suggest that jointly modeling linear structure, nonlinear similarity information, and cross-feature interactions can provide more informative representations for miRNA–disease association prediction. Since several representations in SGMDA are derived from the association matrix, strict fold-specific reconstruction is necessary during cross-validation to prevent held-out associations from contributing to feature construction.

From a biological perspective, the highly ranked candidate associations identified by SGMDA may provide useful clues for further investigation of disease-related miRNAs. The case studies on lung neoplasms and prostate neoplasms showed that many top-ranked predictions were supported by records in external databases, suggesting that the model can prioritize biologically relevant candidate associations. Nevertheless, potential overlap in underlying publications or curated records among HMDD v3.2, dbDEMC v2.0, and miR2Disease should be considered when interpreting these results. Therefore, such database-supported evidence should be regarded as complementary biological support rather than strict independent validation. Moreover, the predicted miRNA–disease associations reflect computationally inferred relationships and should not be interpreted as evidence of biological causality. Further experimental studies are still required to clarify the functional roles and regulatory mechanisms of these candidate miRNAs in specific diseases.

Several methodological limitations should also be considered when interpreting the results. First, experimentally verified negative miRNA–disease associations are scarce and are generally unavailable in existing databases. Therefore, it is difficult to construct a training set containing reliable biological negative samples. In this study, we randomly sampled unlabeled miRNA–disease pairs and treated them as negative samples during model training. However, the absence of an association from the HMDD v3.2 database does not necessarily indicate a true biological negative, because some unlabeled pairs may represent associations that have not yet been experimentally investigated or curated. This strategy may therefore introduce false-negative bias. Future work could consider more reliable negative-sample construction strategies or positive-unlabeled learning methods. In addition, HMDD may be affected by literature and curation bias, because extensively studied miRNAs and diseases are more likely to accumulate reported associations. The dependence of GIP similarity on known interaction profiles may also limit prediction for newly introduced miRNAs or diseases with few or no known associations, resulting in reduced performance under cold-start conditions. Furthermore, the performance of SGMDA depends on the quality and completeness of the available miRNA–disease associations and similarity information. Incomplete or noisy biological data may affect the learned representations and consequently influence prediction performance. Future studies could evaluate the model across additional independent data sources and incorporate more diverse biological information to further improve its robustness and generalizability.

## 6. Conclusions

In this paper, we proposed a novel method named SGMDA for predicting potential miRNA–disease associations. First, we employed SVD to extract the linear structural features from the miRNA–disease association matrix. Simultaneously, we integrated various types of miRNA and disease similarities to construct informative similarity graphs, which were then fed into GAEs to extract nonlinear features. To better fuse these heterogeneous representations, we introduced a contrastive learning strategy that aligned the linear and nonlinear features by minimizing their distance for the same entity while maximizing the distance between different entities. This greatly enhanced the consistency and discriminability of the learned embeddings. Moreover, to capture complex interactions between miRNA and disease features that simple concatenation might overlook, we incorporated a highway network. This structure allowed for effective modeling of deep feature interactions while preserving essential original information, thereby improving the robustness and expressive power of the final feature representations. Extensive experiments demonstrated the effectiveness of SGMDA in predicting potential miRNA–-disease associations. Case studies on lung neoplasms and prostate neoplasms provided additional database-supported evidence for the predictions of SGMDA.

Future work will focus on further improving the generalization and biological interpretability of SGMDA. In particular, we plan to explore more robust learning strategies and incorporate additional biological information, such as gene expression profiles, protein–protein interactions, and epigenetic modifications. Integrating these complementary data sources may provide a more comprehensive biological context for miRNA–disease association prediction and support more reliable identification of potential associations.

## Figures and Tables

**Table 1:** Performance comparison of SGMDA and baseline models based on 5-CV.

	AUC	AUPR	ACC	Precision	Recall	F1	*p*-Value
SGMDA	**0.94 ± 0.01**	**0.93 ± 0.01**	**0.94 ± 0.02**	**0.92 ± 0.01**	**0.91 ± 0.02**	**0.91 ± 0.01**	
AEMDA	0.90 ± 0.02	0.90 ± 0.01	0.90 ± 0.02	0.89 ± 0.01	0.89 ± 0.02	0.89 ± 0.01	$2.7\times 10^{-5}$
VGAEMDA	0.93 ± 0.02	0.91 ± 0.01	0.91 ± 0.02	0.90 ± 0.01	0.90 ± 0.02	0.90 ± 0.01	$1.6\times 10^{-3}$
GONNMDA	0.93 ± 0.01	0.92 ± 0.02	0.93 ± 0.02	0.91 ± 0.01	0.90 ± 0.01	0.90 ± 0.01	$3.0\times 10^{-4}$
SFPred	0.90 ± 0.02	0.91 ± 0.02	0.93 ± 0.01	0.91 ± 0.02	0.90 ± 0.01	0.90 ± 0.02	$4.1\times 10^{-5}$
MAMFGAT	0.92 ± 0.02	0.91 ± 0.01	0.93 ± 0.01	0.91 ± 0.01	0.90 ± 0.02	0.90 ± 0.02	$4.8\times 10^{-4}$

Note: Bold values indicate the best performance for each evaluation metric.

**Table 2:** Performance comparison of SGMDA and baseline models based on global LOOCV.

	AUC	AUPR	ACC	Precision	Recall	F1	*p*-Value
SGMDA	**0.95 ± 0.01**	**0.94 ± 0.02**	**0.94 ± 0.02**	**0.93 ± 0.01**	**0.92 ± 0.02**	**0.92 ± 0.01**	
AEMDA	0.90 ± 0.01	0.90 ± 0.02	0.91 ± 0.01	0.90 ± 0.02	0.90 ± 0.01	0.90 ± 0.02	$3.3\times 10^{-5}$
VGAEMDA	0.91 ± 0.02	0.91 ± 0.01	0.92 ± 0.01	0.91 ± 0.02	0.91 ± 0.01	0.91 ± 0.01	$2.5\times 10^{-5}$
GONNMDA	0.93 ± 0.01	0.93 ± 0.01	0.93 ± 0.02	0.92 ± 0.01	0.91 ± 0.02	0.91 ± 0.01	$1.8\times 10^{-3}$
SFPred	0.92 ± 0.02	0.92 ± 0.01	0.93 ± 0.02	0.91 ± 0.02	0.91 ± 0.01	0.91 ± 0.02	$3.4\times 10^{-4}$
MAMFGAT	0.92 ± 0.02	0.92 ± 0.01	0.93 ± 0.02	0.92 ± 0.01	0.91 ± 0.02	0.90 ± 0.01	$5.3\times 10^{-4}$

Note: Bold values indicate the best performance for each evaluation metric.

**Table 3:** Performance comparison of SGMDA and baseline models based on M-CV.

	AUC	AUPR	ACC	Precision	Recall	F1	*p*-Value
SGMDA	**0.92 ± 0.01**	**0.91 ± 0.02**	**0.92 ± 0.02**	**0.90 ± 0.01**	**0.90 ± 0.01**	**0.90 ± 0.01**	
AEMDA	0.88 ± 0.01	0.89 ± 0.01	0.88 ± 0.01	0.87 ± 0.01	0.87 ± 0.02	0.88 ± 0.02	$4.0\times 10^{-5}$
VGAEMDA	0.89 ± 0.02	0.89 ± 0.01	0.91 ± 0.02	0.88 ± 0.01	0.89 ± 0.01	0.88 ± 0.01	$9.2\times 10^{-5}$
GONNMDA	0.91 ± 0.01	0.90 ± 0.02	0.93 ± 0.01	0.89 ± 0.01	0.89 ± 0.01	0.88 ± 0.02	$7.7\times 10^{-3}$
SFPred	0.90 ± 0.02	0.89 ± 0.02	0.91 ± 0.02	0.89 ± 0.02	0.88 ± 0.01	0.88 ± 0.01	$6.8\times 10^{-4}$
MAMFGAT	0.90 ± 0.02	0.89 ± 0.01	0.90 ± 0.02	0.89 ± 0.01	0.88 ± 0.01	0.88 ± 0.01	$5.2\times 10^{-4}$

Note: Bold values indicate the best performance for each evaluation metric.

**Table 4:** Performance comparison of SGMDA and baseline models based on D-CV.

	AUC	AUPR	ACC	Precision	Recall	F1	*p*-Value
SGMDA	**0.90 ± 0.01**	**0.89 ± 0.02**	**0.90 ± 0.02**	**0.89 ± 0.01**	**0.88 ± 0.01**	**0.88 ± 0.01**	
AEMDA	0.86 ± 0.01	0.86 ± 0.01	0.85 ± 0.01	0.85 ± 0.01	0.86 ± 0.02	0.86 ± 0.02	$3.2\times 10^{-5}$
VGAEMDA	0.87 ± 0.02	0.87 ± 0.01	0.86 ± 0.02	0.86 ± 0.01	0.87 ± 0.01	0.86 ± 0.01	$4.7\times 10^{-4}$
GONNMDA	0.88 ± 0.01	0.87 ± 0.02	0.88 ± 0.01	0.87 ± 0.01	0.86 ± 0.01	0.86 ± 0.02	$4.5\times 10^{-3}$
SFPred	0.86 ± 0.02	0.86 ± 0.02	0.87 ± 0.02	0.86 ± 0.02	0.86 ± 0.01	0.86 ± 0.01	$5.5\times 10^{-5}$
MAMFGAT	0.86 ± 0.02	0.87 ± 0.01	0.86 ± 0.02	0.86 ± 0.01	0.87 ± 0.01	0.86 ± 0.01	$8.1\times 10^{-5}$

Note: Bold values indicate the best performance for each evaluation metric.

**Table 5:** Parameter settings of SGMDA.

Parameter	Value
Similarity threshold	0.5
Linear representation dimension	64
Nonlinear representation dimension	64
Node-level contrastive temperature *T*	0.07
Pair-level contrastive temperature $\tau_{\mathrm{HW}}$	0.05
Learning rate	0.001
Dropout	0.3
Training epochs	200
Number of trees in RF	100
Random seed in RF	42

**Table 6:** Performance comparison of SGMDA and ablated models based on 5-CV.

	AUC	AUPR	ACC	Precision	Recall	F1
SGMDA	**0.94 ± 0.01**	**0.93 ± 0.01**	**0.94 ± 0.02**	**0.92 ± 0.01**	**0.91 ± 0.02**	**0.91 ± 0.01**
SGMDA-LF	0.93 ± 0.01	0.92 ± 0.02	0.93 ± 0.01	0.90 ± 0.01	0.90 ± 0.01	0.90 ± 0.02
SGMDA-NF	0.91 ± 0.02	0.90 ± 0.01	0.91 ± 0.02	0.89 ± 0.01	0.89 ± 0.02	0.88 ± 0.01
SGMDA-SVD	0.93 ± 0.02	0.92 ± 0.01	0.93 ± 0.02	0.90 ± 0.01	0.90 ± 0.02	0.90 ± 0.01
SGMDA-AE	0.93 ± 0.02	0.92 ± 0.01	0.93 ± 0.02	0.90 ± 0.02	0.90 ± 0.01	0.90 ± 0.02
SGMDA-CL	0.91 ± 0.01	0.90 ± 0.02	0.91 ± 0.02	0.89 ± 0.02	0.89 ± 0.01	0.89 ± 0.02
SGMDA-HN	0.92 ± 0.02	0.91 ± 0.01	0.92 ± 0.01	0.90 ± 0.01	0.90 ± 0.01	0.89 ± 0.02

Note: Bold values indicate the best performance for each evaluation metric.

**Table 7:** Top 50 potential miRNAs associated with lung neoplasms.

miRNA	Evidence	miRNA	Evidence
hsa-mir-98	d; m	hsa-mir-660	d
hsa-mir-95	d; m	hsa-mir-16	d; m
hsa-mir-99a	d; m	hsa-mir-545	d
hsa-mir-93	d; m	hsa-mir-610	d
hsa-mir-944	d	hsa-mir-630	d
hsa-mir-31	d; m	hsa-mir-769	unconfirmed
hsa-mir-7	d; m	hsa-mir-520h	d
hsa-mir-574	d	hsa-mir-149	d
hsa-mir-605	d	hsa-mir-205	d; m
hsa-mir-570	d	hsa-mir-557	d
hsa-mir-96	d; m	hsa-mir-526b	d
hsa-mir-548j	unconfirmed	hsa-mir-522	d
hsa-mir-92a	d	hsa-mir-564	d
hsa-mir-629	d	hsa-mir-122	d
hsa-mir-641	d	hsa-mir-1973	d
hsa-mir-638	d	hsa-mir-339	d; m
hsa-mir-558	d	hsa-mir-429	d; m
hsa-mir-652	d	hsa-mir-4326	d
hsa-mir-671	d	hsa-mir-4302	d
hsa-mir-9	d; m	hsa-mir-423	m
hsa-mir-592	d	hsa-mir-873	d
hsa-mir-452	d	hsa-mir-1298	d
hsa-mir-937	d	hsa-mir-145	d; m
hsa-mir-365a	d	hsa-mir-141	d; m
hsa-mir-614	d	hsa-mir-143	d; m

d: dbDEMC v2.0 database; m: miR2Disease database.

**Table 8:** Top 50 potential miRNAs associated with prostate neoplasms.

miRNA	Evidence	miRNA	Evidence
hsa-mir-221	d; m	hsa-mir-155	d
hsa-mir-126	d; m	hsa-mir-122	unconfirmed
hsa-mir-150	d	hsa-mir-146a	m
hsa-mir-183	d; m	hsa-mir-181b	d; m
hsa-mir-223	d; m	hsa-mir-196a	d
hsa-mir-29c	d	hsa-mir-92a	unconfirmed
hsa-mir-200b	unconfirmed	hsa-mir-206	d
hsa-mir-23a	d; m	hsa-mir-19a	d
hsa-let-7a	d; m	hsa-mir-19b	d; m
hsa-let-7e	d	hsa-mir-15b	d
hsa-mir-148a	m	hsa-mir-1	d
hsa-mir-214	d; m	hsa-mir-222	d; m
hsa-mir-30c	d; m	hsa-mir-199a	d; m
hsa-mir-101	d; m	hsa-mir-17	m
hsa-mir-200a	d	hsa-mir-20a	m
hsa-mir-34c	d	hsa-mir-26a	d; m
hsa-mir-31	d; m	hsa-mir-29b	d; m
hsa-mir-133b	d	hsa-mir-15a	d; m
hsa-mir-34b	d	hsa-mir-33a	d; m
hsa-mir-9	d	hsa-mir-21	d; m
hsa-let-7c	d; m	hsa-mir-181a	d; m
hsa-mir-16	d; m	hsa-mir-34a	d; m
hsa-mir-192	d	hsa-mir-133a	d
hsa-mir-143	d; m	hsa-mir-195	d; m
hsa-mir-141	m	hsa-mir-29a	d; m

d: dbDEMC v2.0 database; m: miR2Disease database.

**Table 9:** Temporal validation of SGMDA using HMDD v2.0 and v3.2.

Cutoff	Recovered Associations	Precision@K
Top 100	92	92.00%
Top 500	459	91.80%
Top 1000	903	90.30%

## Data Availability

The public data sources analyzed in this study were HMDD v3.2 and v2.0 [26,47], dbDEMC v2.0 [37], and miR2Disease [38]. The processed datasets and source code used in this study are available at https://github.com/lilei0203/SGMDA (accessed on 1 October 2026).
